# The Prince of Wales's Hospital Fund for London

**Published:** 1899-11-04

**Authors:** 


### Editor's Letter-Box.

[Our Correspondents are reminded that prolixity is a great bar to publication, and that brevity of style and conciseness of statement greatly facilitate early insertion.]

### THE PRINCE OF WALES'S HOSPITAL FUND FOR LONDON.

Dr. W. Knowsley Sibley writes: I have read the letter in a recent issue of The Hospital, signed by the honorary secretaries to the above Fund, correcting some remarks of mine when chairman of the conference convened by the Hospital Reform Association. I am reported to have said, in reference to the principle of inquiring into the means of hospital patients, that I "was sorry that the managers of the Prince of Wales's Fund were rather adverse from inquiry, maintaining that their functions were purely administrative," &c. Although I am in a position to speak from personal experience as to the scope of the inquiries which have been made at hospitals in the past by the visitors of the Fund, my remarks were chiefly made in consequence of a letter published in the *Times* of August 29th, from Sir Trevor Lawrence (a prominent member of the Visiting Committee), in reply to Mr. Lock's letter in the *Times* of August 9th. He said: "With regard to the Visiting Committee, it would in my judgment be a serious mistake for that body to constitute itself, as suggested by Mr. Lock, a tribunal to report on the so-called question of out-patient abuse. The functions of the committee are to inquire into the general administration of the hospitals visited, in regard to buildings, finance, appliances, equipment, and general efficiency. It is undesirable for the committee to enlarge its functions by taking upon itself to interfere with the hospital administration in dealing with patients." Those interested in the success of the Fund and in the question of hospital abuse will welcome the information that notwithstanding Sir Trevor Lawrence's letter to the contrary, the visitors of the Fund do now "specially report not only whether an attempt is made to ascertain the social circumstances of the in-patients, but also whether there is an almoner or inquiry officer for the out-patients." The managers of the Prince of Wales's Fund are indeed to be congratulated on so speedy a reform, and I shall look forward with interest to their next report.

